# Automated Sleep Stages Classification Using Convolutional Neural Network From Raw and Time-Frequency Electroencephalogram Signals: Systematic Evaluation Study

**DOI:** 10.2196/40211

**Published:** 2023-02-10

**Authors:** Shahab Haghayegh, Kun Hu, Katie Stone, Susan Redline, Eva Schernhammer

**Affiliations:** 1 Harvard Medical School Boston, MA United States; 2 Brigham and Women's Hospital Boston, MA United States; 3 California Pacific Medical Center Research Institute San Francisco, CA United States; 4 Medical University of Vienna Vienna Austria

**Keywords:** SleepInceptionNet, polysomnography, PSG, electroencephalogram, EEG, spectrogram, scalogram, short-time Fourier transform, continuous wavelet transform, Welch power spectral density, LeNet, ResNet, Alex Net, inception, convolutional neural network

## Abstract

**Background:**

Most existing automated sleep staging methods rely on multimodal data, and scoring a specific epoch requires not only the current epoch but also a sequence of consecutive epochs that precede and follow the epoch.

**Objective:**

We proposed and tested a convolutional neural network called SleepInceptionNet, which allows sleep classification of a single epoch using a single-channel electroencephalogram (EEG).

**Methods:**

SleepInceptionNet is based on our systematic evaluation of the effects of different EEG preprocessing methods, EEG channels, and convolutional neural networks on automatic sleep staging performance. The evaluation was performed using polysomnography data of 883 participants (937,975 thirty-second epochs). Raw data of individual EEG channels (ie, frontal, central, and occipital) and 3 specific transformations of the data, including power spectral density, continuous wavelet transform, and short-time Fourier transform, were used separately as the inputs of the convolutional neural network models. To classify sleep stages, 7 sequential deep neural networks were tested for the 1D data (ie, raw EEG and power spectral density), and 16 image classifier convolutional neural networks were tested for the 2D data (ie, continuous wavelet transform and short-time Fourier transform time-frequency images).

**Results:**

The best model, SleepInceptionNet, which uses time-frequency images developed by the continuous wavelet transform method from central single-channel EEG data as input to the InceptionV3 image classifier algorithm, achieved a Cohen *κ* agreement of 0.705 (SD 0.077) in reference to the gold standard polysomnography.

**Conclusions:**

SleepInceptionNet may allow real-time automated sleep staging in free-living conditions using a single-channel EEG, which may be useful for on-demand intervention or treatment during specific sleep stages.

## Introduction

Polysomnography (PSG) is the gold standard for assessing sleep quality and diagnosing sleep disorders. PSG sleep staging requires visual inspection of electroencephalogram (EEG), electromyogram, and electrooculogram data, which is time-consuming and labor-intensive, that is, trained technicians may spend hours manually scoring a single night of sleep [1,2]. Thus, the resultant high cost of PSG makes it an unappealing method for longitudinal or population-based sleep studies. The inter- and intrarater variability of PSG scoring is also a concern in manual sleep stage classification [3-5]. In addition, PSG typically requires bulky instrumentation and overnight stays in a sleep laboratory, which may disrupt the natural sleep pattern of patients [2]. To address these limitations, wearable and less obtrusive sleep trackers are more desirable in free-living conditions. One example is actigraphy, which has been widely applied in sleep research owing to its advantages of cost-efficiency and reduced influence on sleep [6]. Actigraphy-based sleep scoring has been further encouraged with the recent use of machine or deep learning algorithms [7,8] or additional physiological parameters such as heart rate variability [9,10]. However, the actigraphy-based sleep scoring algorithms suffer from low specificity, that is, low performance in detecting Wake epochs (between 0.28 and 0.67 [11]), and distinguishing different sleep stages with actigraphy is even more challenging. Hence, alternative noninvasive approaches with better performance are required.

With advances in wearable technology, noninvasive ambulatory recording of single-channel EEG is possible. Sleep staging based on single-channel EEG may be a potential solution to overcome the limitations of PSG and actigraphy. This approach may allow unobstructive, wearable monitoring of sleep stages in free-living conditions, because the setup requires a minimal number of sensors, which can usually be applied by the patients without the help of a technician. Compared with actigraphy, richer temporal information in EEG makes it more suitable for scoring all sleep stages instead of distinguishing only sleep and wake [12-25]. However, no previous studies with the aim of developing single-channel EEG sleep classification have systematically evaluated how different preprocessing methods and EEG channels affect the performance of these algorithms. In addition, most of these studies required a sequence of epochs to score a specific epoch, and thus, are not capable of real-time sleep scoring [20-25]. The aims of this study are to (1) investigate different preprocessing methods of EEG data before importing them into classification algorithms, (2) investigate the performance in sleep stage scoring of available convolutional neural networks using raw or preprocessed EEG data as input, and (3) investigate the effect of different single-channel EEG data and their combination (multichannel) on sleep stage classification performance. The ultimate goal is to identify and test the performance of the best model based on the above findings to check the potential of applying single-channel EEG in real time, that is, immediately after each epoch sleep classification.

## Methods

### Overview

To achieve the aims, we analyzed the overnight PSG recordings collected in the Multi-Ethnic Study of Atherosclerosis (MESA) [26,27]. We used the PSG recordings that were rated as high quality (ie, all channels good for the entire sleep time of >6 hours) by a highly trained team of scorers for the training and testing of all the models. We further evaluated the performance of the best model, SleepInceptionNet, during the transition of sleep stages (or wake) across the overnight period and its generalizability to lower-quality PSG data.

### Data Set

Sleep data collected in the MESA [26,27] were used for this study. The data are freely accessible through the National Sleep Research Resource website through a data use agreement. MESA is a prospective study that investigates the risk factors associated with the development of subclinical and clinical cardiovascular diseases and other health outcomes. In MESA, PSG was conducted between 2010 and 2013 using the Compumedics Somte System (Compumedics Ltd). The system consists of 3 EEG channels (central C4-M1, occipital Oz-Cz, and frontal Fz-Cz leads), bilateral electrooculogram, chin electromyogram, thoracic and abdominal respiratory inductance plethysmography (by auto-calibrating inductance bands), an airflow sensor (by nasal-oral thermocouple and pressure recording from a nasal cannula), electrocardiogram sensors, leg movement sensors, and finger pulse oximetry. All studies were scored using published guidelines for sleep staging [26] by trained research polysomnologists who regularly participated in scoring reliability monitoring and retraining. The interclass correlation coefficients for the 3 primary scorers for sleep staging were 0.96 for Wake (after onset of sleep), 0.86 for N1, 0.63 for N2, 0.81 for N3, and 0.96 for rapid eye movement (REM). Scorers also assigned quality codes to each channel and each study, indicating the time of available signals that were artifact-free and readily scorable as described. As the start and end times of PSG studies were unspecified, the pulse oximetry (SpO2) data of the PSG study were used to determine the actual start and end times, that is, the first and last 5-minute block that contained less than 15 seconds of missing data, poor or marginal quality of SpO2 data (OXSTAT signal).

### Participants

We did not recruit any additional participants for this study. MESA participants with valid PSG data were included in this study. PSG data with high-quality signals, that is, at least 6 hours of valid sleep data in all channels (n=276; 295,013 thirty-second epochs), were used for training and testing the models in this study. In addition, the PSG data of another 607 individuals (642,962 thirty-second epochs) that were not rated as high quality were used to evaluate the generalizability of the best model. Details of the inclusion and exclusion criteria for each of these data sets are presented in Multimedia Appendix 1. Table 1 summarizes the characteristics of the participants. There were no major differences between the characteristics of participants with higher- and lower-quality PSG; however, in the higher-quality data set, the percentage of male participants was slightly higher, with less self-reported troubles in falling asleep, and a higher percentage of African American participants compared with the lower-quality data set.

**Table 1:** Characteristics of the study population.

Characteristics			Higher-quality data (n=276)		Lower-quality data (n=607)		Total (n=883)
Age (years), mean (SD)			67.9 (8.5)		68.9 (8.8)		68.6 (8.7)
**Sex, n (%)**							
	Male	140 (50.7)		269 (44.3)		409 (46.3)	
	Female	136 (49.3)		338 (55.7)		474 (53.7)	
**Race, n (%)**							
	White, Caucasian	103 (37.3)		282 (46.5)		385 (43.6)	
	Chinese American	38 (13.8)		58 (9.6)		96 (10.9)	
	Black, African American	83 (30.1)		138 (22.7)		221 (25)	
	Hispanic	52 (18.8)		129 (21.3)		181 (20.5)	
**Diagnosed with insomnia by a physician, n (%)**							
	Yes	12 (4.4)		27 (4.4)		39 (4.4)	
	No	264 (95.6)		580 (95.6)		844 (95.6)	
**Diagnosed with sleep apnea by a physician, n (%)**							
	Yes	14 (5.1)		43 (7.1)		57 (6.5)	
	No	262 (94.9)		564 (92.9)		(93.5)	
**Diagnosed with restless legs by a physician, n (%)**							
	Yes	11 (4)		21 (3.5)		32 (3.6)	
	No	265 (96)		586 (96.5)		851 (96.4)	
**Trouble falling asleep in the past 4 weeks, n (%)**							
	No, not in the past 4 weeks	161 (58.3)		322 (53.1)		483 (54.7)	
	Yes, less than once a week	33 (12)		66 (10.9)		99 (11.2)	
	Yes, 1 or 2 times a week	40 (14.5)		104 (17.1)		144 (16.3)	
	Yes, 3 or 4 times a week	25 (9.1)		57 (9.4)		82 (9.3)	
	Yes, 5 or more times a week	17 (6.2)		58 (9.6)		75 (8.5)	
**Chronotype, n (%)**							
	Definitely a morning type	96 (34.8)		225 (37.1)		321 (36.4)	
	Rather more a morning than an evening type	73 (26.5)		158 (26)		231 (26.2)	
	Rather more an evening than a morning type	39 (14.1)		64 (10.5)		103 (11.7)	
	Definitely an evening type	33 (12)		89 (14.7)		122 (13.8)	
	Neither a morning nor an evening type	35 (12.7)		71 (11.7)		106 (12)	

### Data Preprocessing

**Figure 1:**
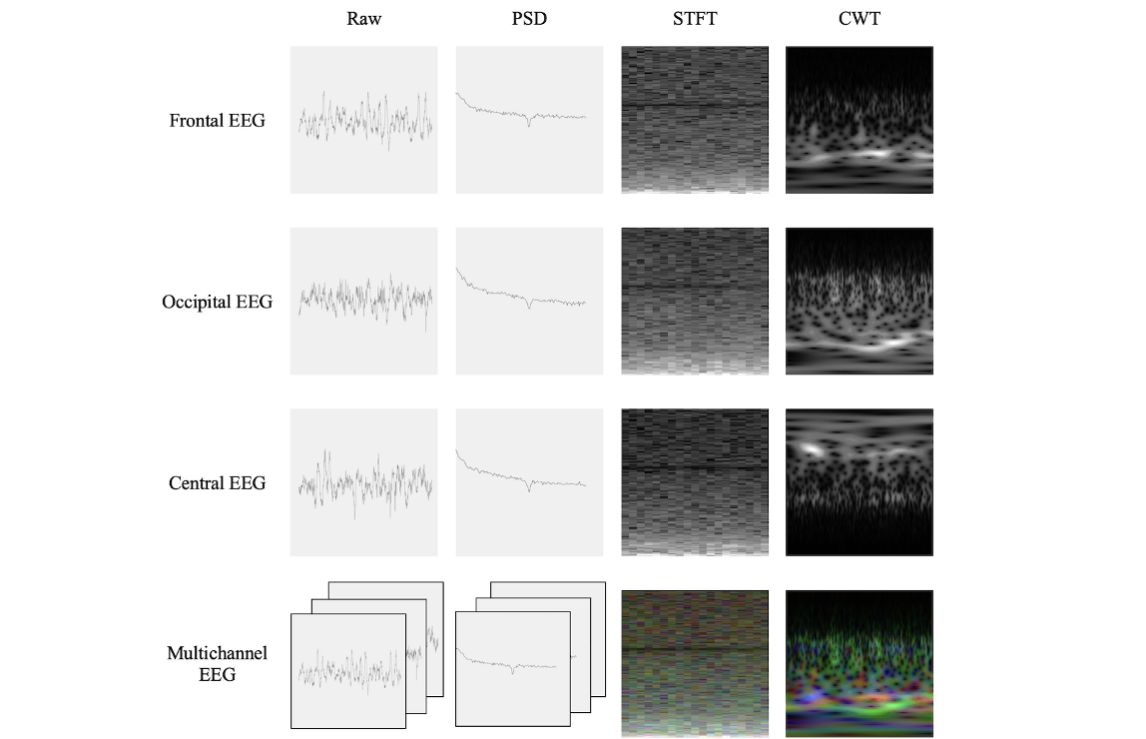
Example of raw 30-second electroencephalogram (electroencephalogram [EEG] data and corresponding processed signals). Left panel: raw EEG signal where the x-axis is associated with the time (second) and the y-axis is associated with the amplitude (mV). The second panel from the left: Welch polysomnography (PSD) of the raw EEG, where the x-axis is associated with the frequency (Hz), and the y-axis is associated with the power. Third panel from the left: short-time Fourier transform of the raw EEG, where the x-axis is associated with the time (seconds), and the y-axis is associated with the frequency (Hz). Right panel: continuous wavelet transform of the raw EEG, where the x-axis is associated with the time (second), and the y-axis is associated with the frequency (Hz). CWT: continuous wavelet transform; PSD: power spectral density; STFT: short-time Fourier transform.

A total of 4 types of EEG data, as follows, were used to derive the inputs for the neural networks: (1) single-channel frontal (Fz-Cz) EEG, (2) single-channel occipital (Oz-Cz) EEG, (3) single-channel central (C4-M1) EEG, and (4) the data of these 3 channels together (multichannel EEG). For each type of data, four different methodologies were used to generate the inputs: (1) no preprocessing (raw EEG), (2) power spectral density (PSD) of EEG, (3) short-time Fourier transform (STFT) of EEG, and (4) continuous wavelet transform (CWT) of EEG. Thus, 16 different sets of inputs were tested separately in this study (Figure 1).

#### Raw EEG

Each raw single- and multichannel EEG signal was divided into 30-second epochs, and all epochs were provided directly as inputs to the neural networks. In this methodology, the input to the networks was a 1D time series, with the shape of (t,1) for single-channel data and (t,3) for multichannel data, where *t*=7680 (30 seconds × 256 data/second) is the number of data points in each 30-second epoch.

#### Welch PSD Estimate

PSD provides the power distribution of the EEG series in the frequency domain [28]. The PSD was generated by the Welch method using a 2-second moving window length with a 0.5-second overlap between moving windows. Using this methodology, the input to the networks was a 1D sequence of data, with the shape of (i,1) for single-channel data and (i,3) for multichannel data, where i=257 is the length of the data sequence (frequencies) in each 30-second epoch.

#### Short-Time Fourier Transform

The spectrograms were generated using a 2-second moving window length, with a 0.5-second overlap between moving windows. Using this methodology, the input to the networks was a 2D image with the shape of (x,y,1), that is, gray image, for single-channel data, and (x,y,3), that is, color image for the multichannel data, where x=256 pixels and y=256 pixels are the width (associated with time) and height (associated with frequency) of the images, respectively.

#### Continuous Wavelet Transform

Scalograms were generated using the Morse wavelet. Using this methodology, the network input has the same shape as that explained in the STFT section.

### Convolutional Neural Networks

To classify the raw and preprocessed data, 2 types of convolutional neural networks were used. Sequential classifiers were used for the sequential data (ie, raw EEG and PSD), whereas image classifiers were used for the 2D data (ie, CWT and STFT).

#### 1D Convolutional Neural Networks (Sequential Classifiers)

A total of 7 classical time series deep neural networks as follows were pre-evaluated on a portion of PSD and raw EEG data of the training data set (n=100, randomly selected participants from the training data set): Fully Convolutional Neural Network (FCN), Residual Network (t-ResNet), Encoder, Multi-Scale Convolutional Neural Network, Time Le-Net (t-LeNET), Multi-Channel Deep Convolutional Neural Network, and Time Convolutional Neural Network. Then, the top 4 networks with the best performance were selected for further training and evaluation using the entire training and test data sets. The network architectures of the top 4 classifiers are presented in Multimedia Appendix 2.

#### 2D Convolutional Neural Networks (Image Classifiers)

A total of 16 available image classifier convolutional neural networks (ie, Xception, VGG16, NASANetLarge, NASANetMobile, ResNet50, ResNet50V2, ResNet101V2, ResNet152V2, DenseNet121, DenseNet169, DenseNet201, InceptionV3, InceptionResNetV2, MobileNetV2, EfficientNetV0, and AlexNet) were pre-evaluated using a portion of the CWT and STFT training data set (n=100, randomly selected participants from training data set). The top 4 models with the best performances were selected for further evaluation using the entire training and test data sets. The network architectures of the top 4 classifiers are presented in Multimedia Appendix 3.

### Training the Models

A 5-fold cross-validation strategy on the PSG data of 276 participants was used to compare the sleep staging performance of different convolutional neural networks, preprocessing methods, and channels of EEG data. Then, the best neural network, preprocessing method, and channel of EEG data were chosen to generate a model, which we named SleepInceptionNet. To train and evaluate the performance of SleepInceptionNet, the PSG data of 276 participants were divided into a test set of 82 participants (30% of the whole data set) and a training and validation set of 194 participants (70% of the whole data set). The test data sets were not used for any training and tuning purposes and were only used to evaluate the performance of the models. In addition, the trained parameters in the cross-validation were not used in the SleepInceptionNet training. As the distribution of sleep stages was heavily imbalanced, the random undersampling method was used to balance the class distribution in the training and validation set (the distribution of wake-sleep stages before undersampling was 30.3% Wake, 9.1% N1, 39.0% N2, 8.2% N3, and 13.4% REM). Undersampling was performed by sampling uniformly at random from the classes with a larger number of epochs (Wake, N2, N3, and REM) to make them the same size as the number of epochs in class N1. Cross entropy was used as the loss function for the classification task, the gradient descent algorithm (learning rate=0.01, momentum=0.9, and decay=1e-6) was used to optimize the neural networks, and the model weights were identified based on minimum validation loss to avoid overfitting. To prevent overfitting in 2D classifiers, data augmentation techniques, that is, horizontal shifting (range −0.4 to 0.4), horizontal flipping, and shearing (range −0.2 to 0.2), were used on the training data sets. The neural networks were generated and trained using TensorFlow (version 2.3.0) [29,30] and the Keras library (version 2.4.0) [31] in Python (version 3.7).

### Performance Evaluation

The sleep staging performance (5-stage classification: Wake, N1, N2, N3, and REM) of different models, preprocessing methods, and channels of EEG data were evaluated using 5-fold cross-validation by Cohen *κ* metric. Cohen *κ* measures the agreement between the algorithm and the ground truth, accounting for the possibility of agreement by chance:



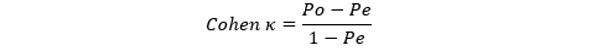



where *P_o_* is the observed agreement, and *P_e_* is the probability of agreement by chance. To evaluate the performance of the SleepInceptionNet model in further detail, recall (sensitivity), specificity, precision, *F*_1_-score, and accuracy were calculated for each class and overall:



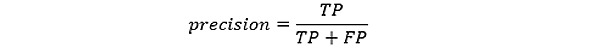





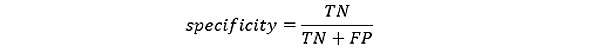





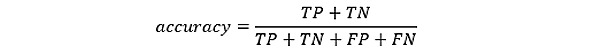





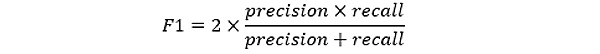





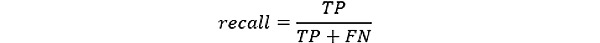



where TP is the true positive (number of epochs correctly scored by the algorithm), TN is the true negative (number of epochs correctly identified by the algorithm as not corresponding to the specific sleep stage), FP is the false positive (number of epochs that were incorrectly scored by the algorithm as the specific sleep stage), and FN is the false negative (number of epochs that were not scored as the specific sleep stage that they should have been).

### Statistical Analysis

Mixed-effect models were used to evaluate the significance level of the effects of different preprocessing methods, EEG channels, and convolutional neural networks. The performance of SleepInceptionNet in scoring epochs of the first and second halves of the PSG recording period was compared using 2-sided paired *t* tests. *P*<.05 was considered a statistically significant difference. Statistical analyses were performed using MATLAB (version R2020a; MathWorks).

## Results

### Performance Comparison of Different EEG Channels, Preprocessing Methods, and Convolutional Neural Networks

Using 5-fold cross-validation strategy on 276 high-quality EEG recordings, we evaluated the performances of 64 different combinations of EEG channels (frontal, occipital, central, and all 3 channels together [multichannel EEG]), preprocessing methods (raw EEG, Welch’s PSD estimate, STFT, and CWT), and convolutional neural networks (4 image classifiers: ResNet50, LeNet, InceptionV3, and AlexNet; and 4 sequential classifiers: FCN, t-ResNet, Encoder, and t-LeNet). The performance evaluation was based on the comparison of the resultant scores with the gold standard scores (manually scored by a highly trained scorer team) and was quantified by Cohen *κ* metric. Tables 2 and 3 present the Cohen *κ* agreements (with 95% CI) across 276 participants with high-quality PSG data (using 5-fold cross-validation).

We first considered how preprocessing of the input data affected the overall performance. Between the 2 preprocessing methods that developed time-frequency images, the CWT-based method provided statistically significantly better performance than that based on STFT (mixed-effect model, *P*<.001, average difference in Cohen *κ* values of CWT-based vs STFT-based=0.037). Between the 2 preprocessing methods that developed sequential data, the PSD-based method provided statistically significantly better performance than that based on raw EEG data (mixed-effect model, *P*<.001, average difference in Cohen *κ* values of PSD-based vs raw EEG−based=0.045). In addition, the CWT-based method provided statistically significantly better performance than the PSD-based method (mixed-effect model, *P*<.001, average difference in Cohen *κ* values of CWT-based vs PSD-based=0.044).

Among the 4 different image classifier algorithms, InceptionV3 and ResNet showed statistically significantly better performance than the average performance of the image classifier algorithms (mixed-effect model; *P*<.001). The performance of InceptionV3 was statistically significantly better than that of ResNet (mixed-effect model, *P*=.007, average difference in Cohen *κ* values of Inception V3 vs ResNet=0.009). Among the sequential 1D classifiers, Encoder and tResNet showed statistically significantly better performances than the average performance of the sequential classifier algorithms (mixed-effect model; *P*<.001), and the performance of Encoder was statistically significantly better than that of tResNet (mixed-effect model, *P*<.001, average difference in Cohen *κ* values of Encoder vs tResNet=0.027).

**Table 2:** Cohen *κ* agreements with ground truth manually scored polysomnography in 5-class sleep staging of different combinations of electroencephalogram (EEG) channels, preprocessing methods (continuous wavelet transform [CWT] and short-time Fourier transform [STFT]), and 2D convolutional neural networks (5-fold cross-validation on 276 participants with higher-quality polysomnography).

Input data and EEG channel			2D convolutional neural networks (image classifiers), mean (95% CI)						
			ResNet50		LeNet		InceptionV3		AlexNet
**CWT**									
	Frontal	0.705 (0.695-0.714)		0.655 (0.639-0.671)		0.712 (0.701-0.723)		0.690 (0.675-0.705)	
	Occipital	0.691 (0.679-0.702)		0.665 (0.642-0.688)		0.700 (0.684-0.717)		0.692 (0.675-0.709)	
	Central	0.715 (0.704-0.727)		0.689 (0.673-0.705)		0.733 (0.718-0.749)		0.709 (0.696-0.722)	
	Multichannel	0.766 (0.757-0.775)		0.713 (0.696-0.729)		0.770 (0.750-0.790)		0.755 (0.740-0.769)	
**STFT**									
	Frontal	0.668 (0.649-0.687)		0.597 (0.586-0.608)		0.676 (0.648-0.703)		0.628 (0.595-0.661)	
	Occipital	0.659 (0.628-0.690)		0.629 (0.602-0.656)		0.668 (0.649-0.686)		0.624 (0.600-0.647)	
	Central	0.702 (0.670-0.733)		0.647 (0.628-0.666)		0.707 (0.680-0.735)		0.670 (0.638-0.702)	
	Multichannel	0.738 (0.718-0.758)		0.693 (0.676-0.709)		0.751 (0.731-0.772)		0.714 (0.693-0.734)	

**Table 3:** Cohen *κ* agreements with ground truth manually scored polysomnography in 5-class sleep staging of 64 different combinations of electroencephalogram channels, preprocessing methods (raw electroencephalogram [EEG] signal and Welch power spectral density [PSD]), and 1D convolutional neural networks (5-fold cross-validation on 276 participants with higher-quality polysomnography).

Input data and EEG channel			1D convolutional neural networks (sequential classifiers), mean (95% CI)						
			t-ResNet		t-LeNet		Encoder		FCN^a^
**Raw EEG signal**									
	Frontal	0.628 (0.588-0.668)		0.487 (0.457-0.516)		0.681 (0.665-0.697)		0.510 (0.491-0.528)	
	Occipital	0.662 (0.630-0.694)		0.547 (0.530-0.563)		0.669 (0.663-0.675)		0.555 (0.519-0.590)	
	Central	0.692 (0.682-0.701)		0.515 (0.495-0.535)		0.707 (0.678-0.737)		0.601 (0.565-0.637)	
	Multichannel	0.717 (0.692-0.743)		0.567 (0.533-0.601)		0.750 (0.737-0.763)		0.644 (0.625-0.662)	
**PSD**									
	Frontal	0.626 (0.609-0.643)		0.612 (0.596-0.629)		0.658 (0.637-0.679)		0.571 (0.541-0.601)	
	Occipital	0.645 (0.628-0.663)		0.654 (0.643-0.665)		0.681 (0.668-0.694)		0.630 (0.614-0.645)	
	Central	0.681 (0.659-0.704)		0.688 (0.675-0.701)		0.706 (0.693-0.718)		0.644 (0.610-0.679)	
	Multichannel	0.736 (0.719-0.754)		0.698 (0.679-0.717)		0.751 (0.732-0.770)		0.676 (0.652-0.701)	

^a^FCN: Fully Convolutional Neural Network.

Next, we considered the differences in performance using different EEG channels. Among all the single-channel results, the central EEG channel (C_4_-M_1_) provided statistically significantly better results than those based on the other single channels (mixed-effect model, 0.024 higher Cohen *κ* values than the average of the results from 3 single-channel results, 0.044 higher Cohen *κ* values than the frontal EEG channel, and 0.027 higher Cohen *κ* values than the occipital EEG channel; *P*<.001 for all the comparisons). The multichannel EEG provided statistically significantly better results than the central EEG channel (mixed-effect model, *P*<.001, average difference in Cohen *κ* values of multichannel vs central EEG channel=0.040).

Overall, among the single-channel EEG algorithms, the InceptionV3 algorithm using time-frequency images based on the CWT of the central channel EEG data provided the best performance in terms of agreement with the ground truth manually scored PSG. We call this model SleepInceptionNet.

### Performance of the SleepInceptionNet Algorithm

Using the test data set of high-quality PSG recordings (n=82), we further evaluated the performance of SleepInceptionNet for each specific wake or sleep stage more carefully. Multimedia Appendix 4 shows the confusion matrix, and Table 4 and Multimedia Appendix 5 present the average with 95% CI across participants (ie, the metric was calculated for each participant and then averaged across participants) and the overall (ie, all the epochs of all the participants were put together and then the metric was calculated) performance indexes. The SleepInceptionNet model showed 0.705 (SD 0.077) Cohen *κ* agreement with manually scored PSG. The worst performance of SleepInceptionNet was in detecting stage N1, and the best performance was in detecting Wake epochs. For these incorrect classifications, Wake epochs were occasionally detected as stage N1 (approximately 6%) and REM (approximately 3%), rarely as stage N2 (approximately 1%), and almost never as stage N3 (<0.01%); stage N1 was most often detected as stage N2 (approximately 19%), REM (approximately 18%), and Wake (approximately 9%) and almost never as stage N3; stage N2 was occasionally detected as stage N3 (approximately 11%), N1 (10%), and REM (5%) and rarely as Wake (approximately 1%); stage N3 was most often confused with stage N2 (approximately 15%) and almost never with other stages (<0.5%), and REM epochs were occasionally scored as stage N1 (approximately 8%) and N2 (approximately 5%) and rarely as Wake (approximately 2%).

**Table 4:** Per class performance (averaged across participants) of SleepInceptionNet using central electroencephalogram channel (C4-M1) data (in a test set of 82 participants with higher-quality polysomnography preprocessed with continuous wavelet transform method).

	Precision, mean (95% CI)	Recall (sensitivity), mean (95% CI)	Specificity, mean (95% CI)	Accuracy, mean (95% CI)	*F*_1_-score, mean (95% CI)
Wake	0.942 (0.928-0.955)	0.886 (0.866-0.906)	0.977 (0.971-0.983)	0.954 (0.948-0.960)	0.909 (0.896-0.923)
N1	0.422 (0.396-0.449)	0.523 (0.496-0.551)	0.921 (0.911-0.931)	0.883 (0.873-0.894)	0.452 (0.432-0.473)
N2	0.865 (0.842-0.888)	0.724 (0.704-0.744)	0.936 (0.927-0.944)	0.856 (0.847-0.865)	0.783 (0.765-0.801)
N3	0.560 (0.490-0.630)	0.842 (0.828-0.856)	0.953 (0.945-0.961)	0.943 (0.936-0.950)	0.636 (0.617-0.656)
REM	0.723 (0.692-0.754)	0.851 (0.822-0.880)	0.946 (0.938-0.954)	0.933 (0.926-0.940)	0.764 (0.740-0.788)
Weighted average of all stages	0.832 (0.822-0.842)	0.786 (0.782-0.791)	0.948 (0.943-0.952)	0.902 (0.895-0.908)	0.797 (0.792-0.801)

We also evaluated the performance of SleepInceptionNet for those transitional epochs (ie, the manually scored sleep stage before or after the epoch was different from the current epoch) using the same 82 high-quality recordings. Multimedia Appendix 6 shows the confusion matrix, and Multimedia Appendices 7 and 8 present the overall and average (with 95% CI) across participants’ performance. The average Cohen *κ* agreement across participants for the transition epochs was 0.486 (SD 0.095), statistically significantly lower than that for all epochs (paired *t* test; difference=0.219, SD 0.074; *P*<.001).

To explore whether the performance of SleepInceptionNet changed across the overnight period, we compared the results obtained from the first and second halves of the overnight period using the same 82 high-quality EEG recordings (Multimedia Appendices 9 and 10). Paired *t* tests showed that the performance for the first half of the recording period was statistically significantly better than that for the second half, as consistently indicated by higher precision (first half: 0.807, SD 0.066; second half: 0.780, SD 0.090; *P*=.01), higher sensitivity (first half: 0.713, SD 0.084; second half: 0.665, SD 0.110; *P*=.001), higher specificity (first half: 0.932, SD 0.036; second half: 0.909, SD 0.056; *P*<.001), higher accuracy (first half: 0.867, SD 0.051; second half: 0.834, SD 0.066; *P*<.001), and higher *F*_1_-score (first half: 0.728, SD 0.081; second half: 0.692, SD 0.102; *P*=.009) in the first half period.

### Generalizability of the SleepInceptionNet on Lower-Quality Data

We further evaluated the performance of SleepInceptionNet in sleep classification of those lower-quality PSG recordings (see the confusion matrix in Multimedia Appendix 11). Table 5 and Multimedia Appendix 12 present the average (with 95% CI) across participants and the overall performance of SleepInceptionNet on lower-quality PSG data (n=607). Using this data set, SleepInceptionNet showed a Cohen *κ* agreement of 0.673 (SD 0.114) with the ground truth manually scored PSG.

Consistent with the results of 82 high-quality EEG recordings, the performance of SleepInceptionNet in these EEG recordings of lower-quality was also reduced statistically significantly for the transitional epochs, ie, the average Cohen *κ* agreement across participants was 0.464 (SD 0.091; paired *t* test; reduction=0.209, SD 0.072; *P*<.001; see details in Multimedia Appendices 13-15). Similarly, the performance for the first half of the overnight period was statistically significantly better than that for the second half as indicated by higher values of weighted average precision, sensitivity, specificity, accuracy, and *F*_1_-score in the first half (0.848, SD 0.061; 0.792, SD 0.085; 0.946, SD 0.045; 0.900, SD 0.054; and 0.807, SD 0.072) than those in the second half (0.810, SD 0.062; 0.740, SD 0.111; 0.926, SD 0.041; 0.870, SD 0.060; and 0.765, SD 0.083; all *P*<.001; Multimedia Appendices 16 and 17).

**Table 5:** Per class performance (averaged across participants) of SleepInceptionNet using central electroencephalogram channel (C4-M1) data (from 607 participants with lower-quality polysomnography, preprocessed with continuous wavelet transform method).

	Precision, mean (95% CI)	Recall (sensitivity), mean (95% CI)	Specificity, mean (95% CI)	Accuracy, mean (95% CI)	*F*_1_-score, mean (95% CI)
Wake	0.924 (0.914-0.933)	0.875 (0.868-0.883)	0.962 (0.954-0.969)	0.938 (0.932-0.944)	0.891 (0.883-0.898)
N1	0.396 (0.385-0.406)	0.502 (0.490-0.513)	0.921 (0.917-0.924)	0.883 (0.879-0.887)	0.429 (0.420-0.438)
N2	0.859 (0.851-0.867)	0.704 (0.694-0.714)	0.928 (0.924-0.932)	0.843 (0.839-0.848)	0.769 (0.762-0.776)
N3	0.545 (0.521-0.569)	0.819 (0.801-0.836)	0.947 (0.943-0.951)	0.938 (0.935-0.941)	0.593 (0.572-0.613)
REM	0.696 (0.681-0.711)	0.797 (0.781-0.813)	0.948 (0.945-0.951)	0.930 (0.927-0.933)	0.725 (0.713-0.738)
Weighted average of all stages	0.821 (0.816-0.826)	0.766 (0.760-0.773)	0.940 (0.937-0.943)	0.889 (0.885-0.893)	0.781 (0.775-0.786)

## Discussion

### Main Contribution of This Study

This study evaluated the performance of different methods for automated sleep stage scoring, including single-channel EEG data compared with ground truth manually scored PSG. The main contributions of this study are (1) identifying the best method for preprocessing EEG signals for sleep staging purposes, (2) identifying the best channel of EEG signals for automatic sleep staging, (3) evaluating the performance of different available convolutional neural networks in scoring sleep stages, and (4) introducing and evaluating the algorithm that we identified as best performing (SleepInceptionNet).

### Effect of EEG Data Preprocessing on Sleep Staging Performance

To identify the best method for preprocessing the EEG signal, we applied 4 different methods (PSD, STFT, and CWT methods in addition to raw EEG signals) to develop the input of the neural networks. PSD provides the distribution of power in the frequency components comprising the EEG signal for a specific period. STFT is a time-frequency decomposition method that performs a Fourier transform within a moving window along the time series with some overlap to generate a spectrogram for each epoch of time series data [32]. Unlike the STFT method, the wavelet analyses use a different time window length for each frequency, that is, longer windows applied to lower frequencies and shorter windows applied to higher frequencies [32,33]; therefore, CWT is an effective method for nonstationary signals such as EEG [33]. The use of PSD data slightly improved the performance of the models compared with raw EEG data, and the time-frequency domain data, that is, CWT and STFT, further improved the performance with better results using CWT compared with STFT.

### Effect of Convolutional Neural Network Structure on Sleep Staging Performance

Regarding the structure of the convolutional neural networks, we initially evaluated 16 available image classifiers and 7 available sequential classifiers on a part of the training data set. Then, the top 4 models of each type of classifier (a total of 8 models) were chosen for further evaluation. Finally, these top convolutional neural network structures were trained on the entire training data set without using the available pretrained weights and evaluated on the test data set. Among the image classifier models, ResNet50 and InceptionV3 had better performance than the others, and between the sequential classifiers, tResNet and Encoder performed better than the others.

### Effect of Brain Regions on Sleep Staging Performance

The American Academy of Sleep Medicine manual on PSG recommends the use of 3 EEG derivations as follows for sleep staging: central EEG (C_4_-M_1_, with backup electrodes of C_3_ for C_4_ and M_2_ for M_1_), occipital EEG (O_2_-M_1_ or C_Z_-O_Z_; with backup electrodes of O_1_ for O_2_, M_2_ for M_1_, C_3_ for C_Z_, and O_1_ for O_Z_), and frontal EEG (F_4_-M_1_ or F_Z_-C_Z_; with backup electrodes of F_3_ for F_4_, M_2_ for M_1_, F_PZ_ for F_Z_, and C_3_ for C_Z_). We used each of these EEG derivations separately, that is, single-channel EEG and their combination, that is, multichannel EEG, as inputs to the neural networks for sleep stage classification. In 15 out of the total 16 different preprocessing methods or convolutional neural networks, the central EEG channel provided the best results compared with frontal and occipital EEG in terms of agreement with the gold standard PSG. The better performance of data from a single central lead compared with an occipital or frontal lead may be related to differences in EEG features across channels relevant to distinguishing stages. Alternatively, the ground truth manually scored PSG data were generated by scorers trained to focus predominantly on the central electrode.

### SleepInceptionNet

The model with the highest agreement with the ground truth manually scored PSG (“SleepInceptionNet”) used the InceptionV3 algorithm, with time-frequency images developed by the CWT method from central channel EEG data as input. The lowest sensitivity in detecting different stages by SleepInceptionNet was related to stage N1, a stage for which interhuman agreement is also low, with intraclass correlation in the range of 0.30 to 0.86 [34]. However, in this data set, N1 interscorer reliability was 0.86. This finding is consistent with other automated sleep staging models that use single-channel EEG data [17,23] or multisignal PSG data [21]. A possible explanation is that stage N1 comprises a relatively small proportion of epochs and occurs in the transition from wakefulness to other sleep stages [35]; therefore, there is the possibility of confusion with Wake. In addition, stages N1 and N2 have similar background activity and are distinguished by the presence of K complexes, sleep spindles, or both, and it is impossible to differentiate between stages N1 and N2 based only on background activity [35]. Furthermore, according to the PSG scoring rules, an epoch with low voltage and mixed frequency EEG that could otherwise be identical to N1 should be scored as N2 if the preceding stage is N2 [21]. Finally, REM and N1 share similar background activity; the only difference is the presence of sharp vertex waves in N1 [35].

The distribution of different sleep stages is highly imbalanced in any PSG study owing to the nature of our sleep, that is, fewer N1 and N3 episodes [21,23,34] compared with other sleep stages. To account for class imbalances, we down-sampled classes with a higher frequency in the training set. Using this approach, SleepInceptionNet achieved one of the highest accuracies reported in the literature for detecting stage N1 [12,14-17,20,21,23,36-38]. Note that this down-sampling technique might improve the sensitivity in detecting stages with lower frequencies, but it may lower the sensitivity in detecting the more frequent stages.

SleepInceptionNet performed better in scoring the epochs of the first half of the PSG recording period compared with the second half. This could be explained by different distributions of sleep stages and different numbers of sleep stage transitions between the first and second halves of the PSG recording. Specifically, there were more Wake epochs (with high detection accuracy) in the first half of the PSG recording (approximately 61% of total Wake epochs) than those in the second half; there were fewer N1 epochs (with low detection accuracy [39]) in the first half of the PSG recording (approximately 37% of total N1 epochs) than those in the second half, and there were fewer sleep stage transition epochs (with the lowest detection accuracy) in the first half of the PSG recording (approximately 45% of the total transition epochs) than those in the second half.

SleepInceptionNet has several strengths compared with other sleep staging systems. First, many previous systems used multisignal or multichannel PSG data [1,21,36,40-48]. Although additional signals might improve the classification performance of the models, our goal was to minimize the number of input signals to the model (and therefore the number of sensors) to be able to use the system in free-living conditions with minimum interruption in regular sleep pattern of patients owing to instrumentation. We were able to show that central single-channel EEG performs almost identically to multichannel EEG. Second, most previous models require a sequence of epochs, eg, a number of epochs before and after the epoch to be scored [20-25]. Although such an approach can also improve the model’s performance, especially in transition epochs, it may not be ideal for real-time sleep stage classification, as it can only analyze the data at the end of a respective sleep period. A strength of SleepInceptionNet is its capability for real-time scoring, which could be very useful when there is a need to apply a specific intervention or treatment during a specific sleep stage. Third, the training and test sets were not independent in several of these studies, that is, the data of each participant were used in both the training and test sets [12,13,18,19]. This approach would cause bias in the findings, and the model might not show the same performance when evaluating it on new patients. Therefore, we did not include any epochs of test participants in our training or validation set. Finally, several previous studies used the unbalanced Physionet Sleep-EDF data set to evaluate their models [12-17,19,39,49]. This data set contains a vast number of Wake epochs from hours before and after the actual sleep. This unbalanced number of Wake epochs could cause bias in the overall performance evaluation, because most models performed well in detecting Wake epochs. In this study, we removed data from the beginning and end of PSG records if they were not associated with the actual sleep study.

### Strengths and Limitations

Strengths of our study are (1) use of PSG obtained in home settings (and thus generalizable to future uses of single EEG monitoring), scored by an established sleep reading center with well-defined practices for training and monitoring reliability; (2) availability of information on the signal quality of each PSG study to assess whether the signal quality influenced the performance; (3) relatively large size of the data set; and (4) evaluating the generalizability of the model to lower-quality PSG data. However, limitations of our study should be noted, and they include the following: (1) the relatively old age of participants (mean age of approximately 69 years), thus not covering all age ranges; however, SleepInceptionNet performed well both in higher- and lower-quality data among this older adult population that is more likely to have sleep problems, suggesting the ability of this algorithm to perform well even in other age ranges with fewer sleep problems, and (2) lack of information on the exact start and end time of studies, and therefore, reliance on pulse oximetry signal quality was used as an indicator of start and end time. Additional research on different data sets entailing different age ranges and sleep problems is needed to further evaluate the performance of SleepInceptionNet. In future studies, investigators should explore the performance of different neural networks such as recurrent neural networks or long short-term memory in sleep stage classification.

### Conclusions

This study demonstrated the benefits of using deep neural networks for automatic sleep stage classification. We also found that the time-frequency domain features of EEG enabled better sleep staging classification than raw EEG. The proposed model, SleepInceptionNet, may allow real-time automated sleep staging in free-living conditions.

## Appendix

Multimedia Appendix 1 Inclusion and exclusion criteria.

Multimedia Appendix 2 Network architecture of 1D convolutional neural networks (Residual Network [t-ResNet], Time Le-Net [t-LeNet], Encoder, and Fully Convolutional Neural Network [FCN]).

Multimedia Appendix 3 Network architecture of 2D convolutional neural networks (ResNet50, LeNet, inceptionV3, and AlexNet).

Multimedia Appendix 4 Confusion matrix of SleepInceptionNet versus polysomnography. REM: rapid eye movement.

Multimedia Appendix 5 Overall per class performance of SleepInceptionNet.

Multimedia Appendix 6 Confusion matrix of scored transition epochs by SleepInceptionNet versus polysomnography. REM: rapid eye movement.

Multimedia Appendix 7 Overall per class performance of SleepInceptionNet in transition epochs. REM: rapid eye movement.

Multimedia Appendix 8 Per class performance (averaged across participants) of SleepInceptionNet in transition epochs. REM: rapid eye movement.

Multimedia Appendix 9 Overall per class performance of SleepInceptionNet during the first and second halves of sleep. REM: rapid eye movement.

Multimedia Appendix 10 Per class performance (averaged across participants) of SleepInceptionNet during the first and second half of sleep. REM: rapid eye movement.

Multimedia Appendix 11 Confusion matrix of scored transition epochs by SleepInceptionNet versus polysomnography using lower-quality polysomnography data. REM: rapid eye movement.

Multimedia Appendix 12 Overall per class performance of SleepInceptionNet using lower-quality polysomnography data. REM: rapid eye movement.

Multimedia Appendix 13 Confusion matrix of scored transition epochs by SleepInceptionNet versus polysomnography using lower-quality polysomnography data. REM: rapid eye movement.

Multimedia Appendix 14 Overall per class performance of SleepInceptionNet in transition epochs using lower-quality polysomnography data. REM: rapid eye movement.

Multimedia Appendix 15 Per class performance (averaged across participants) of SleepInceptionNet in transition epochs using lower-quality polysomnography data. REM: rapid eye movement.

Multimedia Appendix 16 Overall per class performance of SleepInceptionNet during the first and second halves of sleep using lower-quality polysomnography data. REM: rapid eye movement.

Multimedia Appendix 17 Per class performance (averaged across participants) of SleepInceptionNet during the first and second half of sleep using lower-quality polysomnography data. REM: rapid eye movement.

## Abbreviations

CWT: continuous wavelet transform
EEG: electroencephalogram
MESA: Multi-Ethnic Study of Atherosclerosis
PSD: power spectral density
PSG: polysomnography
REM: rapid eye movement
STFT: short-time Fourier transform

## Data Availability

The MESA sleep data set can be obtained through a data use agreement from National Sleep Research Resource.

The image classifier algorithms are freely available in the Python Keras library. In addition, access to the other convolutional neural networks’ codes, preprocessing codes, and instructions on their use are available from the corresponding author on request without charge for noncommercial purposes.
