# Supplementary material for: Automated Sleep Stages Classification Using Convolutional Neural Network From Raw and Time-Frequency Electroencephalogram Signals: Systematic Evaluation Study
Source: J Med Internet Res. 2023 Feb 10;25:e40211. doi: 10.2196/40211 (PMC9960035; doi:10.2196/40211)
Supplement: Multimedia Appendix 1 [file jmir_v25i1e40211_app1.pdf]

Multimedia Appendix 1: Inclusion and exclusion criteria

Inclusion criteria for high-quality dataset (n=276):

The data were included if "Overall Study Quality Grade from type II polysomnography" was "Outstanding", i.e., all channels were rated as "good" for at least 6 hours and the entire duration of sleep. The grades of the signals were assigned at the time of scoring.

Inclusion criteria for lower-quality dataset (n=607):

Data were excluded based on the below criteria:

Variable*	Definition	Excluded if the answer was:
Overall5	Overall Study Quality Grade from type II polysomnography	Outstanding
slewake5	Study Scored Sleep / Wake Only (All Sleep Scored as N2 And No Arousals Scored Due to Poor Quality EEG) from type II polysomnography	Yes
arunrel5	Scoring Arousals Unreliable from type II polysomnography	Yes
losoth5	Data Lost During Study from type II polysomnography	Yes
losbeg5	Data Lost at Beginning of Study from type II polysomnography	Yes
losend5	Data Lost at End of Study from type II polysomnography	Yes
m15	Signal Quality Issues Found on M1 Signal from type II polysomnography	Yes
fpz5	Signal Quality Issues Found on Fpz Signal from type II polysomnography	Yes
ref5	Signal Quality Issues Found on Reference / Ground Signal from type II polysomnography	Yes
remarunrel5	Scoring Arousals in REM (Only) Unreliable from type II polysomnography	Yes
remnrempr5	Scoring REM/NREM Unreliable from type II polysomnography	Yes
stg1stg2pr5	Scoring Stage1/Stage2 Unreliable from type II polysomnography	Yes
stg2stg3pr5	Scoring Stage2/Deep Sleep Unreliable from type II polysomnography	Yes
unustgou5	Unusual Staging from type II polysomnography	Yes
wakslepr5	Scoring Stage Wake/Sleep Unreliable from type II polysomnography	Yes

* Names of variables are chosen as they appear in sleepdata.org
