# Supplementary material for: Automated Sleep Stages Classification Using Convolutional Neural Network From Raw and Time-Frequency Electroencephalogram Signals: Systematic Evaluation Study
Source: J Med Internet Res. 2023 Feb 10;25:e40211. doi: 10.2196/40211 (PMC9960035; doi:10.2196/40211)

Multimedia Appendix 2: Network architecture of 1D CNNs (tResNet, tLeNet, Encoder, and FCN)

Network architecture of tResNet

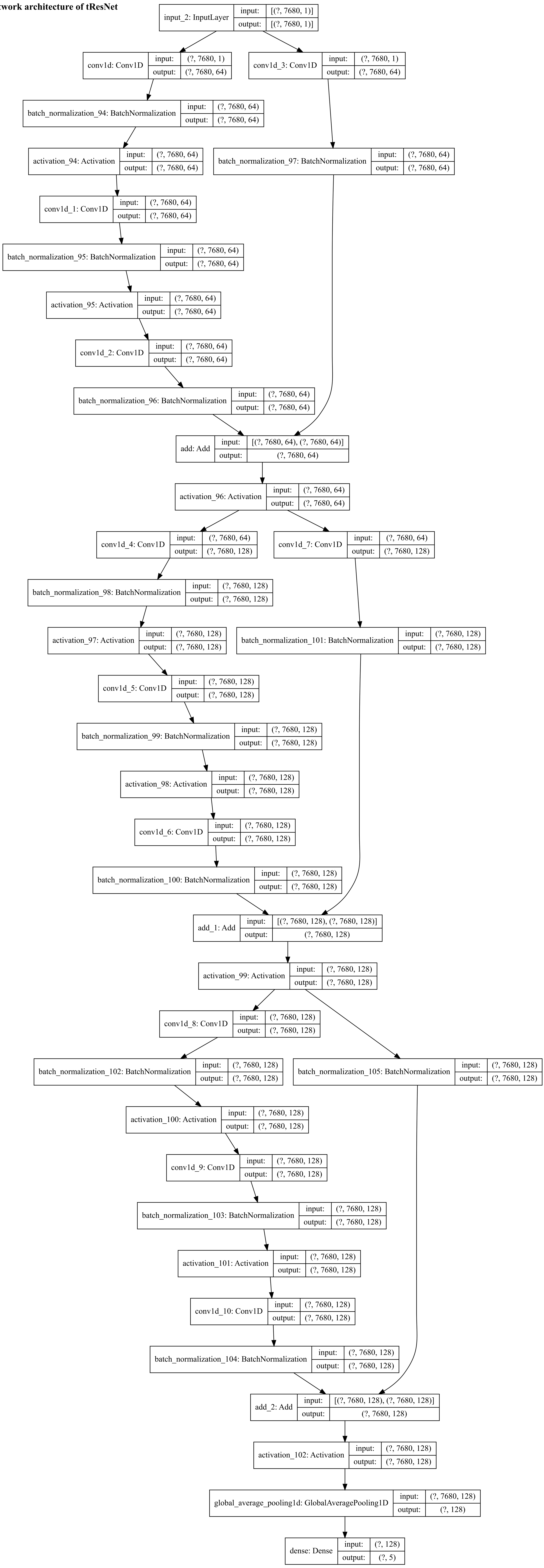

Network architecture of tLeNet

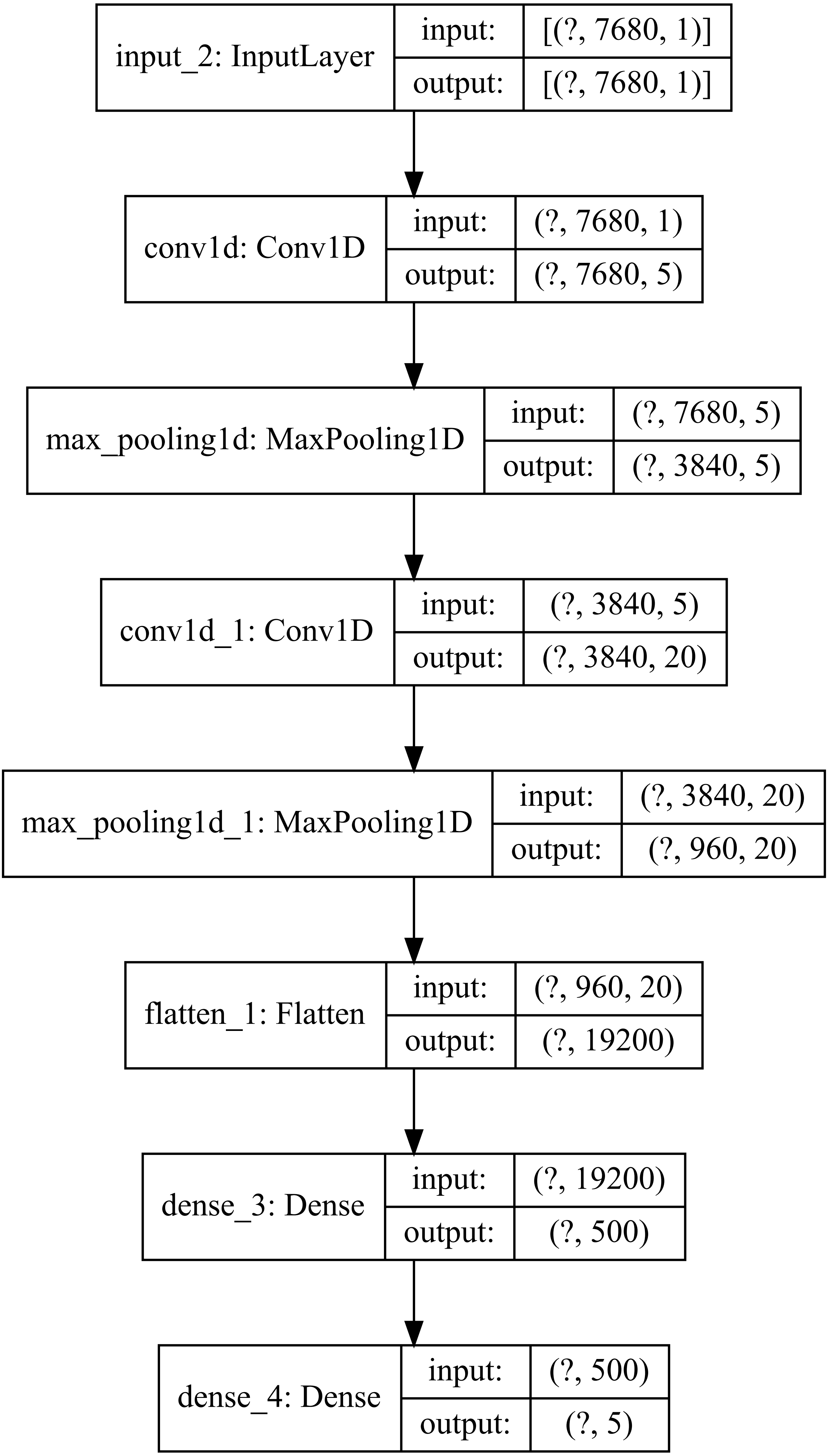

Network architecture of Encoder

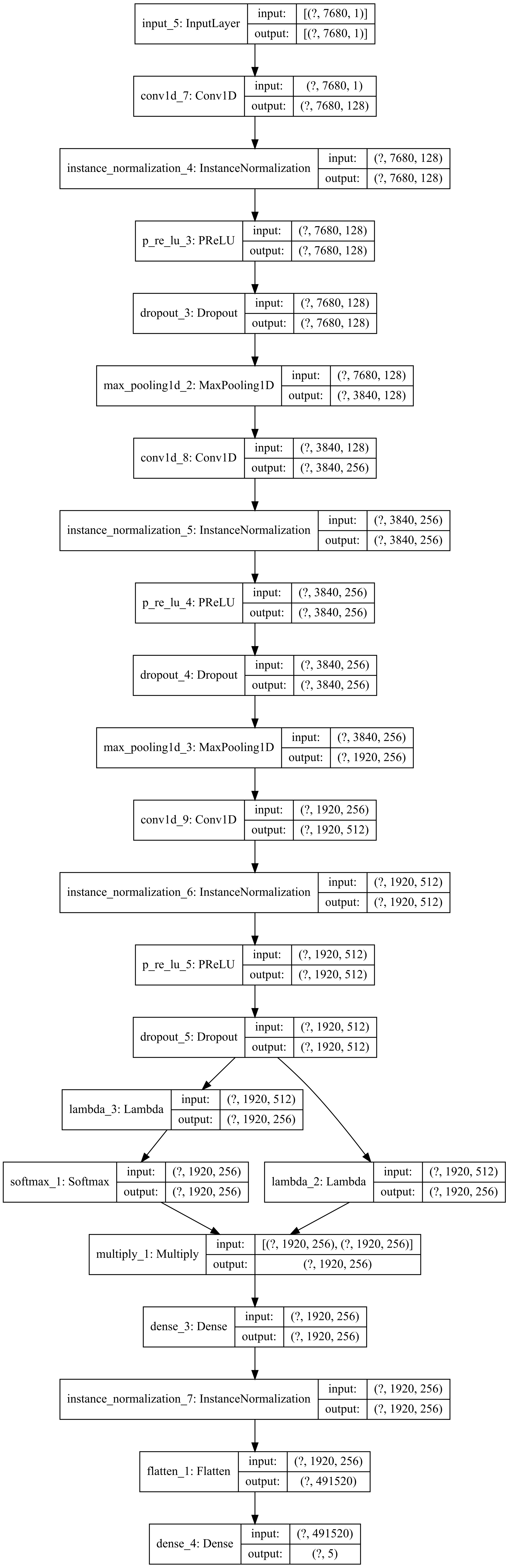

Network architecture of FCN

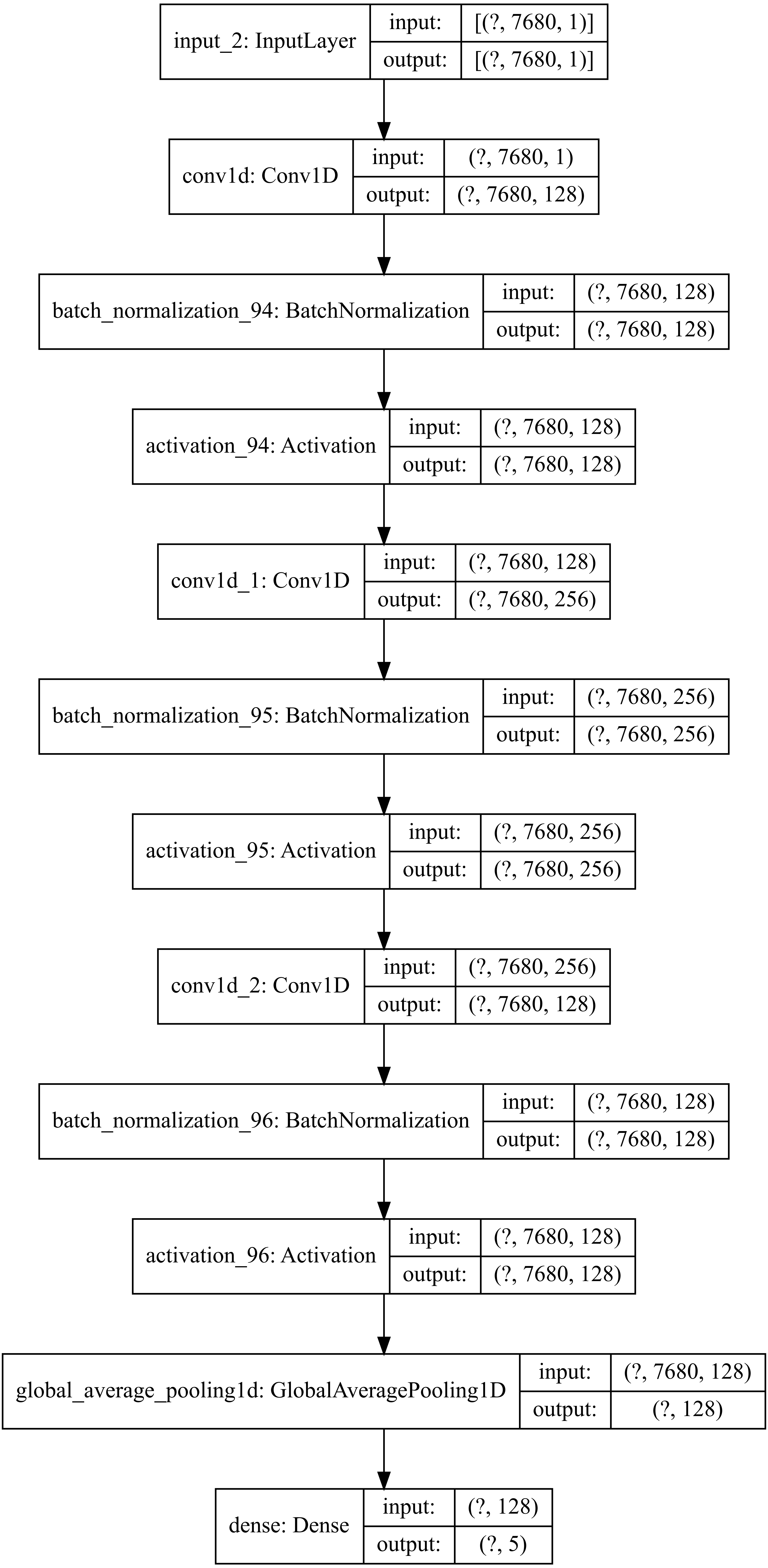

Supplement: Multimedia Appendix 2 [file jmir_v25i1e40211_app2.pdf]
