# Supplementary material for: Automated Sleep Stages Classification Using Convolutional Neural Network From Raw and Time-Frequency Electroencephalogram Signals: Systematic Evaluation Study
Source: J Med Internet Res. 2023 Feb 10;25:e40211. doi: 10.2196/40211 (PMC9960035; doi:10.2196/40211)
Supplement: Multimedia Appendix 4 [file jmir_v25i1e40211_app4.pdf]

Multimedia Appendix 4: Confusion matrix* of scored epochs of test dataset (in a test set data of 82 participants with higher-quality polysomnography (PSG)) by SleepInceptionNet using central electroencephalogram (EEG) channel (C4-M1) data pre-processed with continuous wavelet transform (CWT) method

		SleepInceptionNet				
		Wake	N1	N2	N3	REM
**PSG**	Wake	23560	1687	157	2	809
	N1	846	4734	1656	38	1632
	N2	291	3424	24504	3862	1635
	N3	13	11	1117	6296	14
	REM	231	970	590	11	10251

* Reported as the absolute number of epochs
