# Supplementary material for: Automated Sleep Stages Classification Using Convolutional Neural Network From Raw and Time-Frequency Electroencephalogram Signals: Systematic Evaluation Study
Source: J Med Internet Res. 2023 Feb 10;25:e40211. doi: 10.2196/40211 (PMC9960035; doi:10.2196/40211)
Supplement: Multimedia Appendix 5 [file jmir_v25i1e40211_app5.pdf]

Multimedia Appendix 5: Overall per class performance of SleepInceptionNet using central electroencephalogram (EEG) channel (C4-M1) data (in a test set of 82 participants with higher-quality polysomnography (PSG)), pre-processed with continuous wavelet transform (CWT) method

	**Precision**	**Recall (Sensitivity)**	**Specificity**	**Accuracy**	**F1-score**	**Support***
Wake	0.945	0.899	0.978	0.954	0.921	26215
N1	0.437	0.532	0.923	0.884	0.480	8906
N2	0.874	0.727	0.936	0.856	0.794	33716
N3	0.617	0.845	0.952	0.943	0.713	7451
REM	0.715	0.851	0.946	0.933	0.777	12053
Weighted average of all stages	0.808	0.785	0.950	0.906	0.791	*Total:* 88341

*Support is reported as the absolute number of epochs
