# Supplementary material for: Automated Sleep Stages Classification Using Convolutional Neural Network From Raw and Time-Frequency Electroencephalogram Signals: Systematic Evaluation Study
Source: J Med Internet Res. 2023 Feb 10;25:e40211. doi: 10.2196/40211 (PMC9960035; doi:10.2196/40211)
Supplement: Multimedia Appendix 6 [file jmir_v25i1e40211_app6.pdf]

Multimedia Appendix 6: Confusion matrix* of scored **transition epochs** of test dataset (in a test set data of 82 participants with higher-quality polysomnography (PSG)) by SleepInceptionNet using central electroencephalogram (EEG) channel (C4-M1) data pre-processed with continuous wavelet transform (CWT) method

		SleepInceptionNet				
		Wake	N1	N2	N3	REM
**PSG**	Wake	3067	983	102	1	472
	N1	648	3805	1277	20	1277
	N2	181	1721	5919	1347	665
	N3	11	10	711	1863	9
	REM	53	211	151	6	1547

* Reported as the absolute number of epochs
