# Supplementary material for: Automated Sleep Stages Classification Using Convolutional Neural Network From Raw and Time-Frequency Electroencephalogram Signals: Systematic Evaluation Study
Source: J Med Internet Res. 2023 Feb 10;25:e40211. doi: 10.2196/40211 (PMC9960035; doi:10.2196/40211)
Supplement: Multimedia Appendix 7 [file jmir_v25i1e40211_app7.pdf]

Multimedia Appendix 7: Per class performance in **transition epochs** of SleepInceptionNet using central electroencephalogram (EEG) channel (C4-M1) data (in a test set of 82 participants with higher-quality polysomnography (PSG)), pre-processed with continuous wavelet transform (CWT) method

	**Precision**	**Recall (Sensitivity)**	**Specificity**	**Accuracy**	**F1-score**	**Support***
Wake	0.775	0.663	0.958	0.906	0.715	4625
N1	0.565	0.542	0.846	0.764	0.553	7027
N2	0.725	0.602	0.862	0.764	0.658	9833
N3	0.576	0.715	0.941	0.919	0.638	2604
REM	0.390	0.786	0.899	0.891	0.521	1968
Weighted average of all stages	0.651	0.622	0.886	0.814	0.627	*Total:* 26057

*Support is reported as the absolute number of epochs
