# Supplementary material for: Automated Sleep Stages Classification Using Convolutional Neural Network From Raw and Time-Frequency Electroencephalogram Signals: Systematic Evaluation Study
Source: J Med Internet Res. 2023 Feb 10;25:e40211. doi: 10.2196/40211 (PMC9960035; doi:10.2196/40211)
Supplement: Multimedia Appendix 8 [file jmir_v25i1e40211_app8.pdf]

Multimedia Appendix 8: Per class performance (averaged across participants) in **transition epochs** of SleepInceptionNet using central electroencephalogram (EEG) channel (C4-M1) data (in a test set of 82 participants with higher-quality polysomnography (PSG)), pre-processed with continuous wavelet transform (CWT) method

	**Precision**	**Recall (Sensitivity)**	**Specificity**	**Accuracy**	**F1-score**
Wake	0.790 (0.758, 0.823)	0.656 (0.619, 0.693)	0.948 (0.924, 0.972)	0.897 (0.874, 0.920)	0.697 (0.669, 0.725)
N1	0.555 (0.530, 0.580)	0.519 (0.489, 0.549)	0.837 (0.811, 0.863)	0.758 (0.735, 0.780)	0.526 (0.503, 0.549)
N2	0.723 (0.698, 0.747)	0.589 (0.560, 0.618)	0.849 (0.824, 0.874)	0.753 (0.732, 0.774)	0.641 (0.617, 0.665)
N3	0.571 (0.522, 0.620)	0.735 (0.676, 0.794)	0.924 (0.898, 0.950)	0.903 (0.879, 0.927)	0.604 (0.569, 0.639)
REM	0.404 (0.367, 0.441)	0.763 (0.727, 0.799)	0.885 (0.860, 0.910)	0.878 (0.854, 0.902)	0.505 (0.473, 0.537)
Weighted average of all stages	0.666 (0.647, 0.685)	0.612 (0.592, 0.632)	0.866 (0.844, 0.888)	0.795 (0.774, 0.816)	0.614 (0.593, 0.635)

* Values are reported as mean (95% confidence interval).
