# Supplementary material for: Automated Sleep Stages Classification Using Convolutional Neural Network From Raw and Time-Frequency Electroencephalogram Signals: Systematic Evaluation Study
Source: J Med Internet Res. 2023 Feb 10;25:e40211. doi: 10.2196/40211 (PMC9960035; doi:10.2196/40211)
Supplement: Multimedia Appendix 9 [file jmir_v25i1e40211_app9.pdf]

Multimedia Appendix 9: Overall per class performance of SleepInceptionNet during the first vs. the second half of polysomnography (PSG) recording, using central electroencephalogram (EEG) channel (C4-M1) data (in a test set of 82 participants with higher-quality PSG), pre-processed with continuous wavelet transform (CWT) method

	**Precision**		**Recall (Sensitivity)**		**Specificity**		**Accuracy**		**F1-score**		**Support**	
	1st half	2nd half	1st half	2nd half	1st half	2nd half	1st half	2nd half	1st half	2nd half	1st half	2nd half
Wake	0.963	0.915	0.922	0.863	0.980	0.976	0.959	0.950	0.942	0.888	16057	10158
N1	0.410	0.457	0.556	0.517	0.936	0.910	0.907	0.860	0.472	0.485	3286	5620
N2	0.864	0.883	0.712	0.739	0.942	0.929	0.863	0.849	0.781	0.804	15132	18584
N3	0.669	0.523	0.849	0.837	0.944	0.959	0.933	0.952	0.748	0.643	5175	2276
REM	0.723	0.710	0.848	0.852	0.963	0.929	0.951	0.916	0.780	0.775	4541	7512
Weighted average of all stages	0.829	0.788	0.807	0.763	0.958	0.939	0.918	0.890	0.813	0.770	44191	44150

*Support is reported as the absolute number of epochs
