# Supplementary material for: Automated Sleep Stages Classification Using Convolutional Neural Network From Raw and Time-Frequency Electroencephalogram Signals: Systematic Evaluation Study
Source: J Med Internet Res. 2023 Feb 10;25:e40211. doi: 10.2196/40211 (PMC9960035; doi:10.2196/40211)
Supplement: Multimedia Appendix 10 [file jmir_v25i1e40211_app10.pdf]

Multimedia Appendix 10: Per class performance (averaged across participants) of SleepInceptionNet during the first vs. the second half of polysomnography (PSG) recording, using central electroencephalogram (EEG) channel (C4-M1) data (in a test set of 82 participants with higher-quality PSG), pre-processed with continuous wavelet transform (CWT) method*

	**Precision**		**Recall (Sensitivity)**		**Specificity**		**Accuracy**		**F1-score**	
	1st half	2nd half	1st half	2nd half	1st half	2nd half	1st half	2nd half	1st half	2nd half
Wake	0.960 (0.043)	0.894 (0.124)	0.851 (0.119)	0.763 (0.165)	0.983 (0.026)	0.979 (0.036)	0.942 (0.044)	0.938 (0.052)	0.898 (0.076)	0.809 (0.131)
N1	0.304 (0.145)	0.358 (0.127)	0.500 (0.201)	0.527 (0.209)	0.907 (0.065)	0.874 (0.081)	0.881 (0.059)	0.838 (0.074)	0.358 (0.149)	0.406 (0.136)
N2	0.786 (0.145)	0.807 (0.138)	0.548 (0.165)	0.630 (0.146)	0.920 (0.064)	0.879 (0.105)	0.796 (0.079)	0.776 (0.094)	0.635 (0.148)	0.698 (0.128)
N3	0.427 (0.238)	0.253 (0.224)	0.971 (0.078)	0.944 (0.209)	0.844 (0.086)	0.876 (0.099)	0.863 (0.071)	0.882 (0.088)	0.605 (0.223)	0.417 (0.244)
REM	0.868 (0.205)	0.819 (0.198)	0.537 (0.297)	0.583 (0.285)	0.991 (0.014)	0.969 (0.043)	0.943 (0.040)	0.900 (0.062)	0.640 (0.250)	0.667 (0.220)
Weighted average of all stages	0.807 (0.066)	0.780 (0.090)	0.713 (0.084)	0.665 (0.110)	0.932 (0.036)	0.909 (0.056)	0.867 (0.051)	0.834 (0.066)	0.728 (0.081)	0.692 (0.102)

* Values are reported as mean (95% confidence interval).
