# Supplementary material for: Automated Sleep Stages Classification Using Convolutional Neural Network From Raw and Time-Frequency Electroencephalogram Signals: Systematic Evaluation Study
Source: J Med Internet Res. 2023 Feb 10;25:e40211. doi: 10.2196/40211 (PMC9960035; doi:10.2196/40211)
Supplement: Multimedia Appendix 11 [file jmir_v25i1e40211_app11.pdf]

Multimedia Appendix 11: Confusion matrix* of scored epochs of test dataset (from 607 participants with lower-quality polysomnography (PSG)) by SleepInceptionNet using central electroencephalogram (EEG) channel (C4-M1) data pre-processed with the continuous wavelet transform (CWT) method

		SleepInceptionNet				
		Wake	N1	N2	N3	REM
**PSG**	Wake	176793	13553	1628	238	7210
	N1	6378	31736	12695	142	10610
	N2	7179	24196	175553	30338	11022
	N3	516	66	7717	42356	102
	REM	3246	7397	5534	79	66678

* Reported as the absolute number of epochs
