# Supplementary material for: Automated Sleep Stages Classification Using Convolutional Neural Network From Raw and Time-Frequency Electroencephalogram Signals: Systematic Evaluation Study
Source: J Med Internet Res. 2023 Feb 10;25:e40211. doi: 10.2196/40211 (PMC9960035; doi:10.2196/40211)
Supplement: Multimedia Appendix 12 [file jmir_v25i1e40211_app12.pdf]

Multimedia Appendix 12: Per class performance of SleepInceptionNet using central electroencephalogram (EEG) channel (C4-M1) data (from 607 participants with lower-quality polysomnography (PSG)), pre-processed with continuous wavelet transform (CWT) method

	**Precision**	**Recall (Sensitivity)**	**Specificity**	**Accuracy**	**F1-score**	**Support***
Wake	0.911	0.887	0.961	0.938	0.899	199422
N1	0.412	0.516	0.922	0.883	0.458	61561
N2	0.864	0.707	0.930	0.844	0.778	248288
N3	0.579	0.835	0.948	0.939	0.684	50757
REM	0.697	0.804	0.948	0.930	0.747	82934
Weighted average of all stages	0.791	0.767	0.943	0.895	0.773	*Total:* 642962

*Support is reported as the absolute number of epochs
