# Supplementary material for: Automated Sleep Stages Classification Using Convolutional Neural Network From Raw and Time-Frequency Electroencephalogram Signals: Systematic Evaluation Study
Source: J Med Internet Res. 2023 Feb 10;25:e40211. doi: 10.2196/40211 (PMC9960035; doi:10.2196/40211)
Supplement: Multimedia Appendix 13 [file jmir_v25i1e40211_app13.pdf]

Multimedia Appendix 13: Confusion matrix* of scored **transition epoc**hs of test dataset (in a test set data of 607 participants with lower-quality polysomnography (PSG)) by SleepInceptionNet using central electroencephalogram (EEG) channel (C4-M1) data pre-processed with continuous wavelet transform (CWT) method

		SleepInceptionNet				
		Wake	N1	N2	N3	REM
**PSG**	Wake	21681	7493	797	38	3847
	N1	5165	25987	10479	128	8563
	N2	2710	12297	42799	9817	4598
	N3	236	56	4736	12815	63
	REM	559	1588	1284	24	9639

* Reported as the absolute number of epochs
