# Supplementary material for: Automated Sleep Stages Classification Using Convolutional Neural Network From Raw and Time-Frequency Electroencephalogram Signals: Systematic Evaluation Study
Source: J Med Internet Res. 2023 Feb 10;25:e40211. doi: 10.2196/40211 (PMC9960035; doi:10.2196/40211)
Supplement: Multimedia Appendix 14 [file jmir_v25i1e40211_app14.pdf]

Multimedia Appendix 14: Overall per class performance in **transition epochs** of SleepInceptionNet using central electroencephalogram (EEG) channel (C4-M1) data (in a test set of 607 participants with lower-quality polysomnography (PSG)), pre-processed with continuous wavelet transform (CWT) method

	**Precision**	**Recall (Sensitivity)**	**Specificity**	**Accuracy**	**F1-score**	**Support***
Wake	0.714	0.640	0.944	0.889	0.675	33856
N1	0.548	0.516	0.844	0.756	0.532	50322
N2	0.712	0.593	0.850	0.751	0.647	72221
N3	0.562	0.716	0.941	0.919	0.629	17906
REM	0.361	0.736	0.902	0.891	0.484	13094
Weighted average of all stages	0.630	0.603	0.878	0.803	0.608	*Total:* 187399

*Support is reported as the absolute number of epochs
