# Supplementary material for: Automated Sleep Stages Classification Using Convolutional Neural Network From Raw and Time-Frequency Electroencephalogram Signals: Systematic Evaluation Study
Source: J Med Internet Res. 2023 Feb 10;25:e40211. doi: 10.2196/40211 (PMC9960035; doi:10.2196/40211)
Supplement: Multimedia Appendix 15 [file jmir_v25i1e40211_app15.pdf]

Multimedia Appendix 15: Per class performance (averaged across participants) in **transition epochs** of SleepInceptionNet using central electroencephalogram (EEG) channel (C4-M1) data (in a test set of 607 participants with lower-quality polysomnography (PSG)), pre-processed with continuous wavelet transform (CWT) method*

	**Precision**	**Recall (Sensitivity)**	**Specificity**	**Accuracy**	**F1-score**
Wake	0.787 (0.774, 0.800)	0.644 (0.631, 0.657)	0.942 (0.934, 0.950)	0.889 (0.882, 0.896)	0.679 (0.670, 0.688)
N1	0.538 (0.529, 0.547)	0.501 (0.489, 0.513)	0.845 (0.839, 0.851)	0.759 (0.754, 0.764)	0.508 (0.499, 0.517)
N2	0.722 (0.715, 0.729)	0.587 (0.576, 0.598)	0.843 (0.836, 0.850)	0.747 (0.742, 0.752)	0.637 (0.630, 0.644)
N3	0.578 (0.563, 0.593)	0.739 (0.718, 0.760)	0.934 (0.929, 0.939)	0.914 (0.909, 0.919)	0.602 (0.591, 0.613)
REM	0.390 (0.376, 0.404)	0.707 (0.689, 0.725)	0.898 (0.891, 0.905)	0.887 (0.881, 0.893)	0.475 (0.463, 0.487)
Weighted average of all stages	0.661 (0.656, 0.666)	0.601 (0.595, 0.607)	0.867 (0.863, 0.871)	0.793 (0.789, 0.797)	0.606 (0.600, 0.612)

* Values are reported as mean (95% confidence interval).
