# Supplementary material for: Automated Sleep Stages Classification Using Convolutional Neural Network From Raw and Time-Frequency Electroencephalogram Signals: Systematic Evaluation Study
Source: J Med Internet Res. 2023 Feb 10;25:e40211. doi: 10.2196/40211 (PMC9960035; doi:10.2196/40211)
Supplement: Multimedia Appendix 16 [file jmir_v25i1e40211_app16.pdf]

Multimedia Appendix 16: Overall per class performance of SleepInceptionNet during the first vs. the second half of polysomnography (PSG) recording, using central electroencephalogram (EEG) channel (C4-M1) data (in a test set of 607 participants with lower-quality PSG), pre-processed with continuous wavelet transform (CWT) method

	**Precision**		**Recall (Sensitivity)**		**Specificity**		**Accuracy**		**F1-score**		**Support**	
	1st half	2nd half	1st half	2nd half	1st half	2nd half	1st half	2nd half	1st half	2nd half	1st half	2nd half
Wake	0.949	0.846	0.917	0.834	0.968	0.956	0.948	0.928	0.932	0.840	126697	72722
N1	0.407	0.417	0.523	0.510	0.937	0.907	0.906	0.861	0.458	0.459	24431	37130
N2	0.850	0.876	0.686	0.724	0.938	0.921	0.852	0.836	0.759	0.793	108990	139298
N3	0.615	0.515	0.844	0.816	0.938	0.958	0.928	0.951	0.712	0.631	34090	16667
REM	0.677	0.708	0.817	0.798	0.964	0.931	0.951	0.908	0.740	0.750	27428	55506
Weighted average of all stages	0.815	0.768	0.792	0.742	0.952	0.931	0.910	0.878	0.798	0.749	321636	321323

*Support is reported as the absolute number of epochs
