# Supplementary material for: Automated Sleep Stages Classification Using Convolutional Neural Network From Raw and Time-Frequency Electroencephalogram Signals: Systematic Evaluation Study
Source: J Med Internet Res. 2023 Feb 10;25:e40211. doi: 10.2196/40211 (PMC9960035; doi:10.2196/40211)

Multimedia Appendix 3: Network architecture of 2D CNNs (ResNet50, LeNet, inceptionV3, and AlexNet)

Network architecture of ResNet50

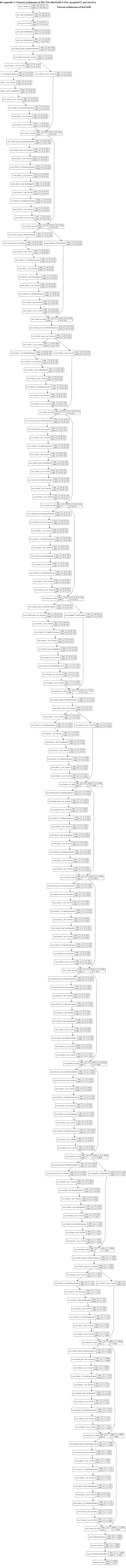

Network architecture of LeNet

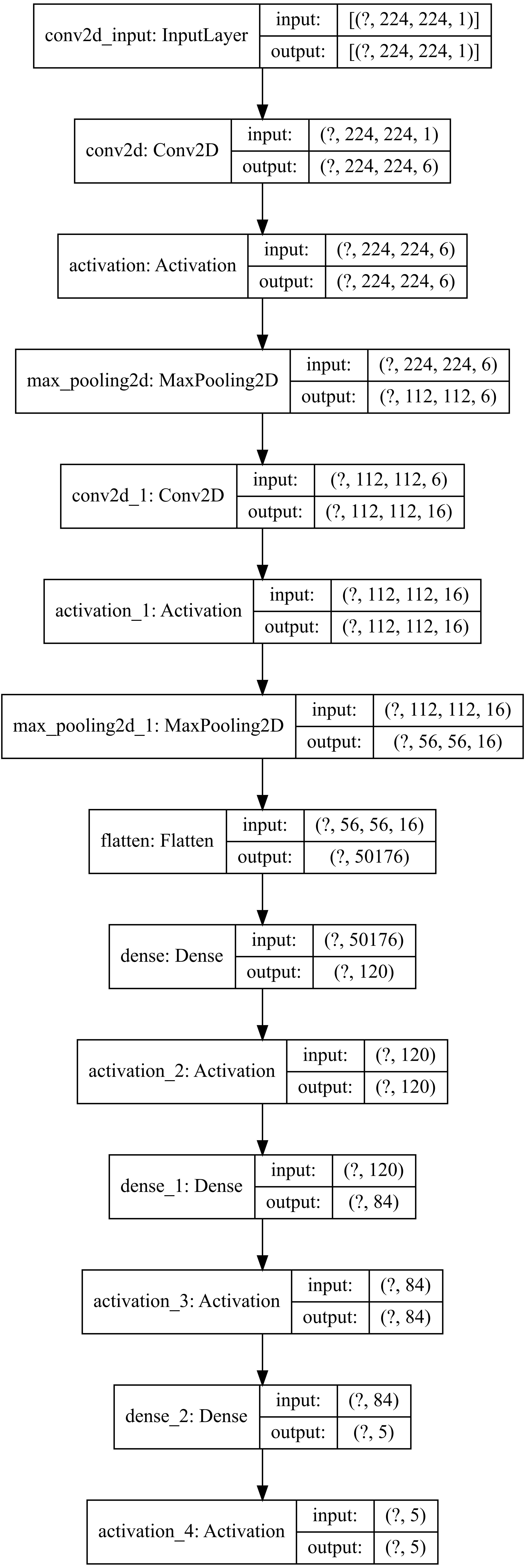

Network architecture of inceptionV3

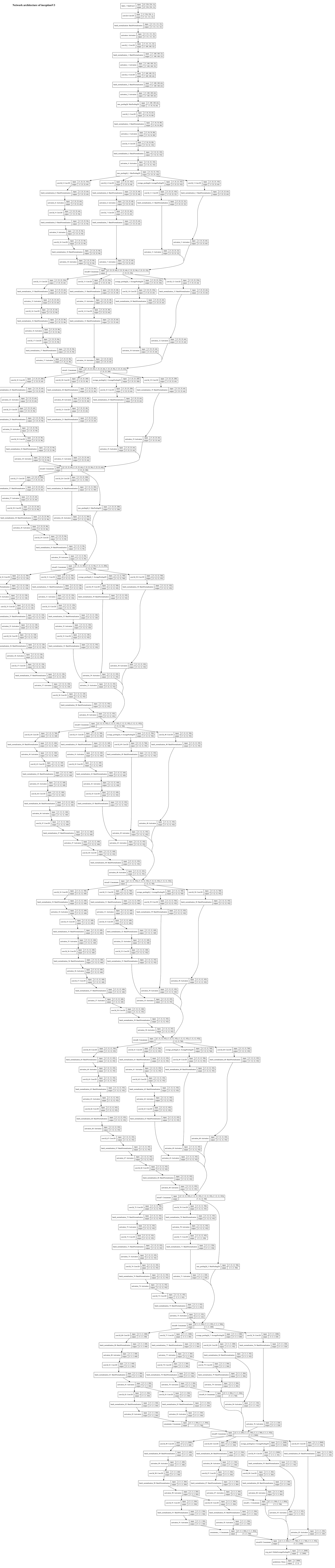

Network architecture of AlexNet

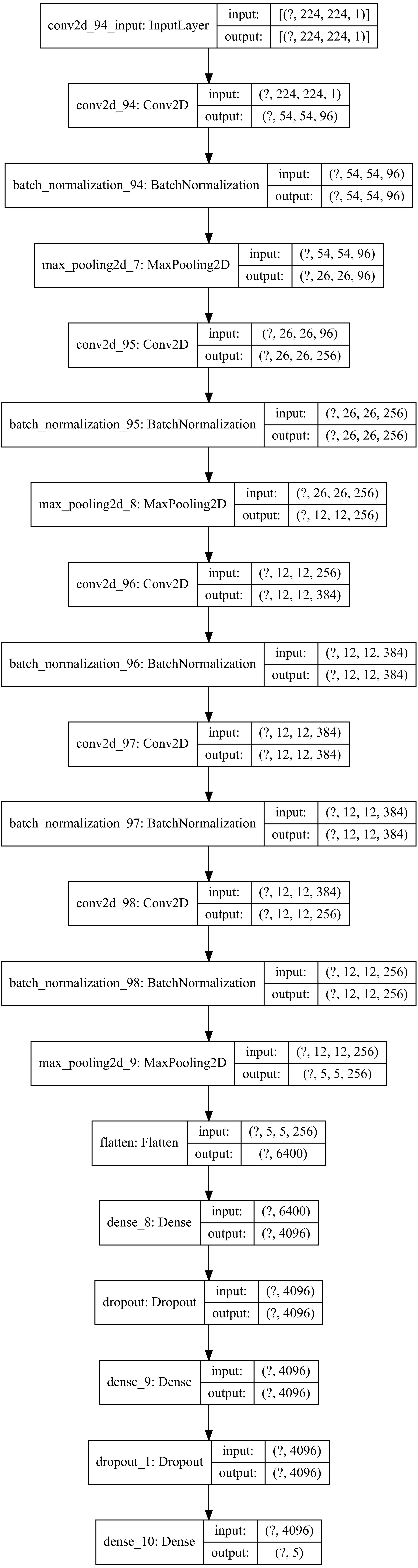

Supplement: Multimedia Appendix 3 [file jmir_v25i1e40211_app3.pdf]
